# Epithelial tumours of the thymus

**DOI:** 10.1054/bjoc.2001.1764

**Published:** 2001-05

**Authors:** P Ruffié, G Gory-Delabaere, B Fervers, J F Regnard, M Resbeut

**Affiliations:** Institut Gustave Roussy, Villejuif; Cancer Research Campaign, Paris; Centre Val d'Aurelle Paul-Lamarque, Montpellier; Centre Léon Bérard, Lyon; Centre Chirurgical Marie Lannelongue, Le Plessis Robinson; Institut Paoli-Calmettes, Marseille, France


*British Journal of Cancer* (2001) **84**(Supplement 2), 51–54
© 2001 FNCLCC
doi: 10.1054/ bjoc.2001.1764, available online at http://www.idealibrary.com on IDEAL®

### Epithelial tumours of the thymus

**P Ruffié[1], G Gory-Delabaere[2,3], B Fervers[2,4], JF Regnard[5] and M Resbeut[6]**

[1]Institut Gustave Roussy, Villejuif; [2]FNCLCC, Paris; [3]Centre Val d'Aurelle Paul-Lamarque, Montpellier; [4]Centre Léon Bérard, Lyon; [5]Centre Chirurgical Marie Lannelongue, Le Plessis Robinson; [6]Institut Paoli-Calmettes, Marseille, France

Epithelial tumours of the thymus, including thymomas and thymic carcinomas are rare tumours (250 new cases a year). Because of its anatomic situation and rarity, this cancer poses special problems in both diagnosis and treatment. Clinical management requires the input of a multidisciplinary team. These guidelines were validated in February 2000 and an update is planned for 2001. A new classification system for thymic tumours was published in 1999. Its place will be considered in the next update.

### Terminology

There is no standard terminology, but it is recommended that the particular characteristics of these tumours are taken into consideration: the term 'encapsulated' (65% of cases) or 'invasive' (35% of cases) should be used in preference to benign or malignant thymoma; the term 'epithelial tumour of the thymus' includes the thymomas and the thymic carcinomas in the same group and excludes benign epithelial cysts, germ cell epithelial tumours (such as teratomas or embryonal carcinomas) and intrathymic parathyroid tumours; the term 'epithelial tumour of the thymus' should be used in preference to 'thymoma'.

### Classification

There is no standard histological classification. Central review of the histological slides by a panel of experienced pathologists is recommended, as well as the routine documentation of any cellular atypia. The classification of Marino and Muller-Hermelink in a simplified form (cortical, medullary and mixed) is the most widely used internationally. It is recommended that this classification is used in conjunction with the more established classifications such as those of Verley or Rosai and Levine.

### Staging

The staging system of Masaoka should be used (standard). The system of the Thymic Tumour Study Group or that of Regnard can also be used (option) as they may be better adapted to treatment strategies. However, these need to be validated against the reference classification of Masaoka, which should therefore still be used outside a trial context.

### Diagnosis

The diagnosis of an epithelial tumour of the thymus is made by the histological examination of a biopsy obtained by anterior mediastinotomy (except in the case of an encapsulated tumour which is usually resectable 'en mass' (standard)). In the case of undifferentiated or lymphocyte-predominant forms, the differential diagnosis must include Hodgkins lymphoma, non-Hodgkins lymphoma or a germ cell tumour (standard). For carcinomas of the thymus, a metastasis from a non-small cell carcinoma must be excluded. A transparietal needle biopsy is an alternative to anterior mediastinotomy (option). A mediastinoscopy is not recommended as it does not provide adequate access to the anterior mediastinal space.

### Pretherapeutic assessment

The standard pretherapeutic investigations are: imaging (chest X-ray (AP and lateral), thoracic CT scan with high abdominal cuts), respiratory function tests full blood count and immunoelectrophoresis of paraproteins to exclude an autoimmune syndrome.

Optional investigations include: thoracic MRI (instead of CT scanning) or a venocavogram if infiltration or compression of vasculature by tumour is suspected; fibreoptic bronchoscopy in cases of suspicion of compression or invasion of the trachea or bronchus; electromyography and auto-antibody screen (for acetylcholine, antithymus, antistriated muscle) if myasthenia gravis is suspected.

### Prognostic factors

The extent of resection and the stage of the disease are the only factors to have unequivocal prognostic value on multivariate analysis (standard).

### TREATMENT MODALITIES

### Surgery

The object is to achieve complete excision of tumour with the thymus and perithymic fat (standard). Sternotomy is the principle route of approach (standard). Videothoracoscopy is at present contraindicated. In advanced stages, the surgery must in all cases preserve the integrity of at least the phrenic nerve (standard).

For very large thymic tumours and/or those extending to the pleura and/or where a pulmonary resection is likely to be necessary, a bilateral anterior thoracotomy with transverse sternotomy can be considered (option). For small-volume thymic tumours in myasthenic patients, a cervicomanubrial approach can be used (option). In the case of ectopic thymomas a posterolateral thoracotomy can be undertaken (option).

### Radiotherapy

There is no standard approach. The recommendations for target volume and dose are as follows.

*Treatment field*
The entire thymic region should be treated including sites of spread (pericardium, large vessels, pleura, lung parenchyma). The fields are defined with the help of pre- and postoperative imaging and also by the operative description and the positioning of radio-opaque clips/markers.

The upper border should be positioned at the level of the cervicothoracic junction. The lower limit should be the mid mediastinum, except in the case of ectopic forms. Irradiation of the supra-clavicular spaces is not recommended as it has not been shown to be useful (level of evidence C).

*Dose*
Following complete resection, a dose of 50–55 Gy, depending on the size of the original tumour, the mediastinal structures involved and the dose that will be delivered to normal tissue. Following incomplete resection treatment should be according to conformational techniques, after consideration of dose–volume histograms both for planning target volume and for critical organs, in particular the lung parenchyma and the spinal cord.

In the absence of neoadjuvant treatment, 50–55 Gy to the target volume with a boost to 60–65 Gy at the level of any residual tumour as identified from the operative report and markers left at the time of operation, should be applied. In the case of a simple biopsy, a dose of 65 Gy is recommended for the entire target volume.

*Scheduling*
Nine to 10 Gy weekly in five sessions. It is recommended that patients be included in therapeutic trials.

### Chemotherapy

Chemotherapy is indicated for those patients presenting with metastatic disease (10%), and for patients with local recurrence or metastases who have already been treated with radiotherapy (standard). Polychemotherapy appears superior to monotherapy (level of evidence C).

The reference combination is the CAP protocol (cyclophosphamide/doxorubicin/cisplatin). In stages IIIA and IIIB, the value of chemotherapy in addition to surgery and radiotherapy has not been proven. The inclusion of patients in prospective studies is recommended in order to demonstrate the efficacy of these combinations, particularly in the neoadjuvant setting.

### THERAPEUTIC STRATEGY

The objective is to achieve complete excision of the tumour with all the thymus and the perithymic fat. It must be carried out by a surgeon who is familiar with the diagnostic and therapeutic constraints of the procedure (standard). Treatment depends on the stage of the disease and the completeness of resection. It can be planned around the three existing classifications.

*Stage IA (encapsulated tumour, without invasion of the capsule)*
Complete resection, no additional treatment (Figure 1).

*Stage IB (encapsulated tumour but with adhesion and/or suspicion of macroscopic invasion of the capsule)*
Complete resection, postoperative radiotherapy at a dose in the order of 50 Gy (Figure 1).

*Stage II (tumour with microscopic invasion into capsule, mediastinal pleura or sub-pleural fat)*
Complete resection, postoperative radiotherapy at a dose in the order of 50–55 Gy (Figure 2).

*Stage III (tumour with macroscopic invasion to lung, superior vena cava, pericardium)*
Complete resection (Regnard stage IIIA disease): postoperative radiotherapy at a dose of at least 55 Gy. Incomplete resection (GETT stage IIIA, Regnard stage IIIB): postoperative radiotherapy at 55–60 Gy with a boost to residual tumour as marked by operative clips. Resection initially impossible (biopsy alone,

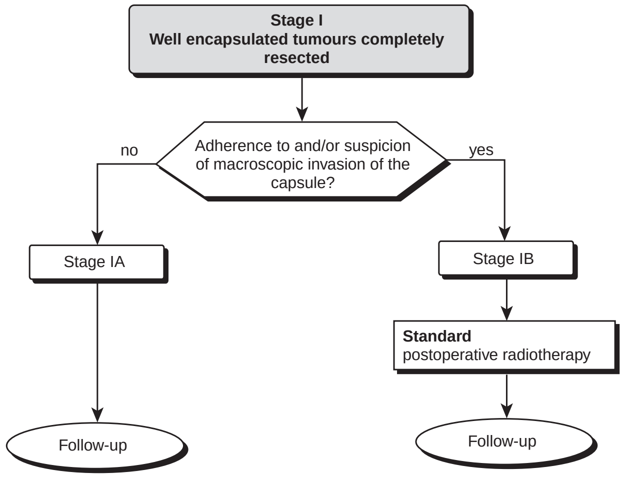

**Figure 1** Treatment of stage I carcinoma of the thymus

© 2001 FNCLCC

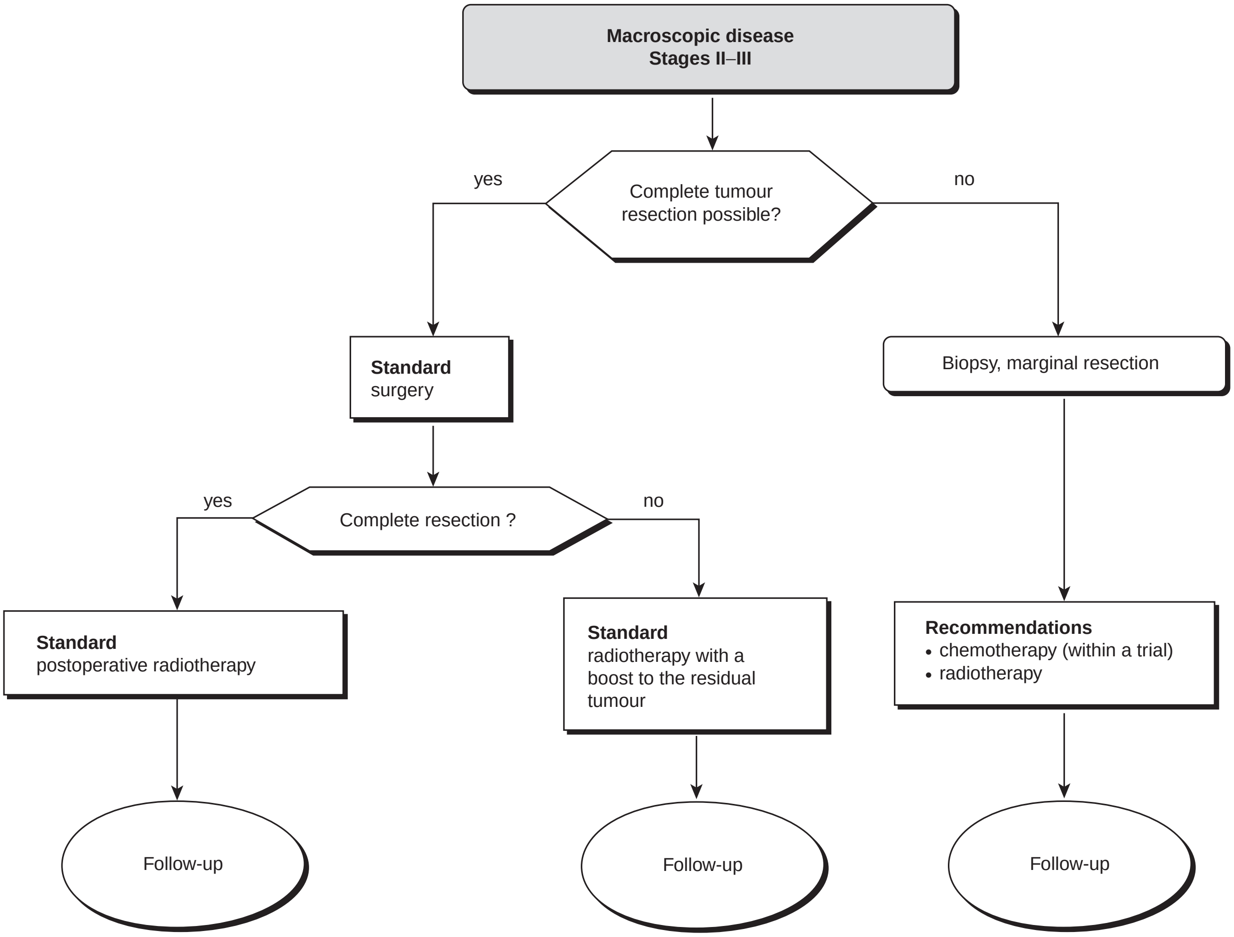

**Figure 2** Treatment of stage II and III disease

GETT and Regnard stage IIIB disease): neoadjuvant chemotherapy followed by resection or radiotherapy. These patients should be included in therapeutic trials (Figure 2).

### *Stage IV disease (mixed population)*

Pleural invasion, completely resected (stage IVA): postoperative radiotherapy to mediastinum and pleura to a dose of 55 Gy according to perioperative markers. Pleural invasion not amenable to surgical excision (Masaoka and GETT stage IVA): neoadjuvant chemotherapy followed by surgery and radiotherapy. Pleural invasion with incomplete surgical excision (Masaoka and GETT stage IVA, Regnard stage IVB) or distant metastases: chemotherapy then surgical re-evaluation and/or subsequent radiotherapy (Figure 3).

### FOLLOW-UP

In the absence of objective data, the frequency of surveillance has not been clearly defined, but it must be continued for at least 15 years in view of the possibility of very late relapses. The appearance of signs and symptoms of an autoimmune syndrome, particularly myasthenia gravis, should result in an early search for recurrence.

### INTERNAL REVIEWERS

T Bachelot (Centre Régional Léon Bérard, Lyon), V Beckendorf (Centre Alexis Vautrin, Vandœuvre-Lès-Nancy), JJ Bretel (Institut Gustave Roussy, Villejuif), Y Bruchon (Centre Georges-François Leclerc, Dijon), D Cowen (Institut Paoli Calmettes, Marseille), G Depadt (Centre Oscar Lambret, Lille), JY Douillard (Centre René Gauducheau, Nantes), J Fraisse (Centre Georges-François Leclerc, Dijon), JL Lagrange (Centre Antoine Lacassagne, Nice) (coordonnateur des SOR pathologies thoraciques), A Lortholary (Centre Paul Papin, Angers), F Mornex (Centre Régional Léon Bérard, Lyon) and P Rebattu (Centre Régional Léon Bérard, Lyon).

### EXTERNAL REVIEWERS

C. Allavena (Centre Catherine de Sienne, Nantes), R Arriagada (IRAM, Santiago – Chili), J Borrelly (CHU-Hôpital de Brabois, Vandœuvre-Lès-Nancy), F Burki (Clinique Pasteur, Toulouse), M Chahinian (Mount Sinaï Medical Center, New-York), E Chirat (Centre de Radiothérapie, Meudon la Forêt), JP Dujols (Centre de Radiothérapie et d'Oncologie Médicale, Pau), A Dujon (Centre Médico-Chirurgical du Cèdre, Bois-Guillaume), E Dulmet (Centre

© 2001 FNCLCC

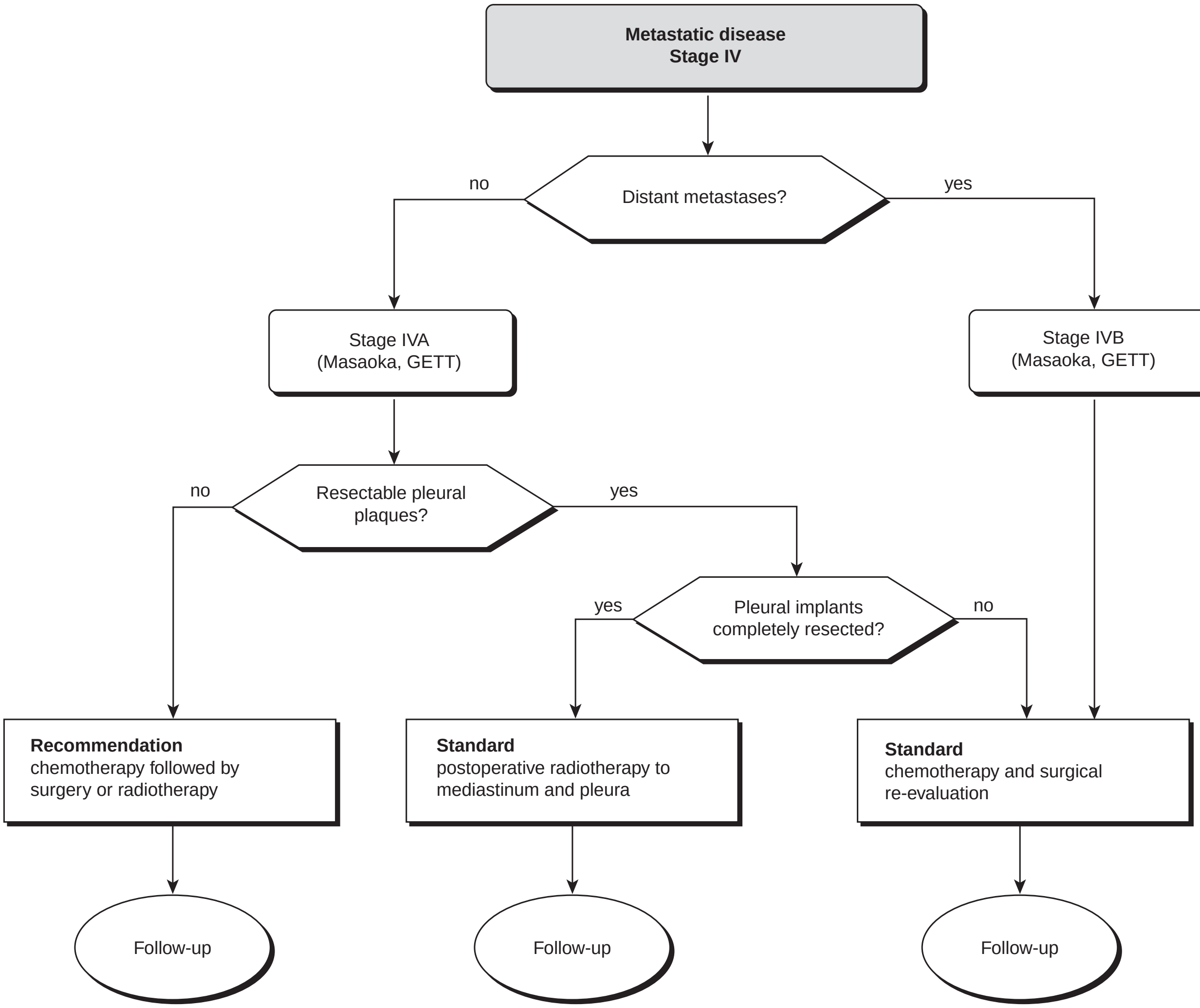

**Figure 3** Treatment of stage IV disease

Chirurgical Marie Lannelongue, Le Plessis Robinson), JP Dumur (Centre Médical des Chartreux, Aix en Provence), JP Dutin (Centre Saint-Michel, La Rochelle), F Economides (Dunkerque), B Eymard (Centre Hospitalier de la Pitié Salpétrière, Paris), F Guichard (Bordeaux), P Martin (Clinique Saint-Jean, Lyon), C Pasteris (Clinique de Savoie, Annemasse), JL Reynoard (Clinique Victor Hugo, Angoulême) and P Vuillemin (Centre de Radiothérapie du Pays d'Aix, Aix en Provence).

*© 2001 FNCLCC*

